# Transformer-based short-term traffic forecasting model considering traffic spatiotemporal correlation

**DOI:** 10.3389/fnbot.2025.1527908

**Published:** 2025-01-23

**Authors:** Ande Chang, Yuting Ji, Yiming Bie

**Affiliations:** ^1^College of Forensic Sciences, Criminal Investigation Police University of China, Shenyang, China; ^2^School of Transportation, Jilin University, Changchun, China

**Keywords:** intelligent transportation system, short-term traffic forecasting, Transformer, traffic spatiotemporal correlation, deep learning

## Abstract

Traffic forecasting is crucial for a variety of applications, including route optimization, signal management, and travel time estimation. However, many existing prediction models struggle to accurately capture the spatiotemporal patterns in traffic data due to its inherent nonlinearity, high dimensionality, and complex dependencies. To address these challenges, a short-term traffic forecasting model, Trafficformer, is proposed based on the Transformer framework. The model first uses a multilayer perceptron to extract features from historical traffic data, then enhances spatial interactions through Transformer-based encoding. By incorporating road network topology, a spatial mask filters out noise and irrelevant interactions, improving prediction accuracy. Finally, traffic speed is predicted using another multilayer perceptron. In the experiments, Trafficformer is evaluated on the Seattle Loop Detector dataset. It is compared with six baseline methods, with Mean Absolute Error, Mean Absolute Percentage Error, and Root Mean Square Error used as metrics. The results show that Trafficformer not only has higher prediction accuracy, but also can effectively identify key sections, and has great potential in intelligent traffic control optimization and refined traffic resource allocation.

## 1 Introduction

Traffic forecasting is a fundamental component of intelligent transportation systems (ITS). The primary goal of traffic forecasting is to identify key factors influencing traffic variation based on historical observations, develop prediction models, and forecast future traffic conditions (Yu, 2021; Rong et al., 2022). Traffic forecasting is typically categorized into short-term and long-term predictions, depending on the forecast horizon. In this study, the focus is on short-term predictions, which generally aim to forecast traffic conditions within the next hour. It is particularly significant in the real-world context of ITS for several reasons (Ji et al., 2023; Li et al., 2025). First, accurate short-term forecasts directly benefit travelers by providing more precise travel time estimates, which help individuals make informed decisions about their departure times and route choices. This can lead to more efficient traffic distribution and reduced overall travel time (Bie et al., 2024; Luo et al., 2024). Furthermore, for transportation operators, effective short-term forecasting enables the implementation of real time management strategies, such as dynamic route guidance. This helps mitigate congestion before it reaches critical levels and reduces the risk of accidents (Sun et al., 2018a,b). However, short-term traffic forecasting also faces specific challenges, particularly due to the stochastic nature of traffic flow and the influence of external factors such as weather, accidents, and special events.

In pursuit of more accurate traffic forecasting accuracy, many methods have been explored. These methods typically take historical traffic data as input or combine it with other actual data sources. Through a variety of means, they mine the characteristics within the traffic flow data to achieve predictions of traffic flow features, such as traffic flow speed or traffic volume. They are mainly divided into two categories: model methods based on linear statistical theory and nonlinear theory. Methods based on linear statistical theory, such as historical mean prediction, time series prediction (Ma et al., 2021; Han, 2024), Kalman filtering prediction (Okutani and Stephanedes, 1984; Zhang et al., 2023), are characterized by their simplicity, ease of implementation, and low computational cost for a single prediction. However, they usually fail to address the uncertainty and nonlinearity of traffic flow, thereby lacking the capability of effective prediction in complex environments. Nonlinear theoretical model-based methods mainly include wavelet analysis (Wang and Shi, 2013; Dong et al., 2021), chaos theory (Shi et al., 2020), neural network, and support vector regression (Omar et al., 2024). Among these, wavelet analysis models and chaos theory can extract nonlinear characteristics and achieve relatively high accuracy, but due to their high complexity, research on traffic forecasting based on these methods is relatively limited (Zhang et al., 2018). Neural network models and models based on support vector regression have rich parameters and strong fitting ability for complex nonlinear relationships, making them the mainstream prediction methods currently employed (Wang et al., 2023; Wang J. et al., 2024).

Early neural network models are essentially shallow neural networks (NN), which were unable to comprehensively extract the fundamental features from traffic data. Therefore, neural network models with multiple hidden layers (MHL), such as Multilayer Perceptron (MLP), have gradually been applied in traffic forecasting (Oliveira et al., 2021). With the increase in model complexity, the network’s ability to extract traffic features enhances, but at the same time, it requires a larger number of training samples and the prediction time per single training also increases. Due to computational limitations, early machine learning algorithms did not demonstrate significant advantages in traffic forecasting problems. In 2006, Hinton et al. introduced the first Deep Learning (DL) paper, highlighting two key insights: deep neural networks with MHL excel at feature learning, providing a more fundamental data representation, and “layer-wise pre-training” effectively mitigates the challenges of training deep networks. The publication of this article sparked the wave of research in DL (Nigam and Srivastava, 2023).

Recurrent Neural Networks (RNN) (Pascanu, 2013), along with variants like Long Short-Term Memory (LSTM) (Schmidhuber and Hochreiter, 1997) and Gated Recurrent Unit (GRU) (Yang et al., 2022), are effective at handling sequential data and conducting complex transformations. These capabilities enable them to capture temporal dependencies in traffic flow, making them ideal for time series forecasting (He et al., 2022). In addition, with the widespread use of surveillance equipment, convolutional neural network (CNN) models, which rely on image data, have been introduced into traffic forecasting (Parishwad et al., 2023). Based on the multilayer convolution structure inherent in CNN models, these models can effectively capture spatial correlation characteristics of traffic flow (Narmadha and Vijayakumar, 2023). On the other hand, graph neural networks (GNNs) models (Scarselli et al., 2008), which are based on graph-structured data, have also been applied to traffic forecasting. GNNs are good at modeling the relationships between different nodes in a traffic network, especially in capturing topological structures and interactions. They are suitable for scenarios where the spatial relationship between roads and intersections plays a vital role. Subsequently, Transformer-based models (Vaswani et al., 2017) have gradually shown great potential in traffic forecasting problems. Compared with other traffic forecasting methods, Transformer can simultaneously focus on different positions of the input sequence through its unique multi-head attention mechanism, thereby more comprehensively capturing long-distance dependencies and complex features in traffic data. In addition, the architecture design of Transformer allows it to perform parallel calculations, greatly improving the training efficiency. Compared with some methods based on CNNs/GNNs, it has obvious speed advantages when processing large-scale traffic data sets, and can adapt to dynamic changes in traffic conditions more quickly, providing a more efficient solution for real time traffic forecasting (Eleonora and Pinar, 2023; Chen et al., 2024; Zoican et al., 2024; Guo B. et al., 2024; Guo X. et al., 2024).

However, existing methods still have limitations. For example, traditional graph-based models may face challenges of high computational complexity due to complex graph convolution operations and strict dependence on road topology. Similarly, in the Transformer’s self-attention, while it typically uses all node information to compute attention weights, the traffic network, composed of roads and intersections, has complex spatial relationships that cannot be captured by a simple linear sequence. As a result, the current approach introduces unnecessary interactions and noise, limiting its ability to fully capture the network’s spatial characteristics. Taking into account the complexity of traffic flow and the limitations of existing methods, the historical traffic flow data sequence and road topology information of traffic nodes are used as the core input data source. A DL framework based on the Transformer encoding module is constructed to achieve accurate prediction of future traffic speed at traffic nodes. Specifically, spatial masks based on spatial topology and travel time are designed. In this way, spatial information is effectively introduced, significantly enhancing the model’s ability to capture spatial relationships in complex urban traffic scenarios and greatly improving traffic flow prediction accuracy. In addition, a streamlined and effective MLP is used to replace the original complex decoding structure of the Transformer. This reduces the computational complexity and the number of network layers while ensuring that the prediction accuracy is not compromised. The main contributions of this work include:

1. Using the road network topology to generate spatial masks, so that the model can take more into account the traffic nodes with spatial connections during feature interaction, which reduces the unnecessary interaction and noise.
2. Introducing a Transformer-based traffic forecasting model, which can effectively handle long-term dependencies in spatiotemporal traffic information and provide more interpretability.
3. Conducting multiple sets of comparative experiments and ablation studies using a large-scale real road network dataset to assess the model’s performance, accuracy, and its internal components.

The remainder of the paper is structured as follows. “Literature review” covers DL-based traffic forecasting methods. “Methodology” introduces the DL framework established in this study. “Experiments” validates the proposed approach with real world datasets. The research conclusions and prospects are presented in “Conclusions.”

## 2 Literature review

As a core part of ITS, traffic flow prediction aims to anticipate traffic conditions, such as traffic flow speed, traffic flow volume, enabling authorities to take preemptive measures and travelers to plan better. However, traffic flow is complex, affected by various factors. Traditional prediction methods struggle to capture its dynamic nature. With computing power growth, machine learning, especially DL, has emerged as a leading solution (Zhu et al., 2021; Mohammadian et al., 2023; Ding et al., 2024; Chen et al., 2024; Wang Q. et al., 2024). Different DL architectures offer unique strengths in handling traffic flow data. RNN and their variants, like LSTM, are designed to handle sequential data, making them suitable for capturing temporal patterns in traffic flow. CNN excel at extracting spatial features, which is vital for understanding the relationships between different traffic nodes (Li et al., 2024a). And Transformer, with its attention mechanism, can model full dependencies, better handling long-range correlations in traffic. Hence, the following sections will explore these three categories of DL-based traffic forecasting methods.

### 2.1 Traffic forecasting based on RNN

RNN and their improved architectures are a highly utilized class of NN in the field of traffic forecasting. Tian and Pan (2015) developed a recursive LSTM model that incorporates three multiplication units in the memory block, allowing for dynamic selection of the optimal time lag from historical input, leading to better prediction accuracy. Zhao et al. (2017) constructed a two-dimensional LSTM network with multiple memory units to facilitate short-term traffic flow forecasting. They also compared the established model with other representative prediction models to verify its effectiveness. Yu et al. (2017) constructed a hybrid deep model based on LSTM for traffic forecasting under extreme conditions and realized the joint simulation of traffic flow states under normal conditions and accident modes. A bidirectional RNN module was used by Liu et al. (2017) to analyze historical traffic data at nodes, uncover periodic traffic flow patterns, and incorporate them into urban traffic forecasting. Fang et al. (2023) reconfigured the loss function in LSTM based on the negative guidance mixed correlation entropy criterion, aiming at the prediction error caused by non-Gaussian noise, and constructed a delta-free LSTM framework for short-term traffic flow prediction.

### 2.2 Traffic forecasting based on CNN

CNNs have been utilized by some researchers for traffic forecasting tasks. They use multilayer convolutional structures and their combined networks to extract the spatiotemporal correlation features of traffic flows. Ma et al. (2022) built a feature selection algorithm based on the combined units of CNN and GRU, and combined the positive and reverse GRU networks to mine the long-distance dependencies in the input information to increase the accuracy of predictions. Wang and Susanto (2023) used CNN to represent and process features such as traffic flow change patterns in different time periods in a way similar to image features, so as to better understand and use the information in time series data to predict traffic flow. However, traditional CNN frameworks are better suited for processing data with uniform size and dimension, typically found in Euclidean structure data. In the context of traffic networks, the road connections between traffic nodes may not be uniformly distributed, and the feature matrix dimensions of nodes may also vary. Therefore, the spatial characteristics learned by CNN may not necessarily represent the optimal features of the traffic network structure. The introduction of graph convolutional networks (GCN) (Kipf and Welling, 2016) has brought breakthroughs in the application of CNN in non-Euclidean structured data (Gong et al., 2023; Guo B. et al., 2024; Guo X. et al., 2024). By using the topological structure information of the graph to adjust the convolution operation, CNN can better adapt to the irregular data distribution and complex node relationships in the traffic network, thereby significantly improving its performance in tasks such as traffic forecasting (Li et al., 2023).

### 2.3 Traffic forecasting based on transformer

Transformer, as one of the variations of DL network architectures, was introduced by Vaswani et al. (2017). It models the full dependencies between inputs and outputs using attention mechanisms. Models and frameworks based on Transformer can better handle long-range dependencies in traffic flow data, exhibiting relatively higher flexibility. Based on the overall architecture of Transformer, Cai et al. (2020) identified the continuous and periodic patterns in traffic time series, modeled the spatial dependence of the road network, and finally verified the model’s impact through two real data sets. Yan et al. (2021) used the combined framework of the global encoder and the global–local decoder to realize the extraction and fusion of global and local traffic flow features and achieved high-precision prediction of urban traffic flow. Chen et al. (2022) constructed a dual-directional spatiotemporal adaptive transformation framework based on codec-decoder structure to address the uneven spatiotemporal distribution in traffic prediction, and verified its effectiveness on four datasets. Wang F. et al. (2024) proposed a comprehensive network based on Transformer and GCN to capture the complex spatiotemporal correlations in metropolitan area networks and achieve more accurate traffic forecasting. The attention distribution in Transformer partly reveals the correlation information of traffic flow across different traffic nodes in spatial and temporal dimensions, improving the model’s interpretability.

Table 1 lists the basic models, input information, datasets used and other key information of some methods. Based on Table 1, it can be seen that most of the early short-term traffic forecasting methods are based on a single detector to obtain time series data, such as traffic volume collected by sensors. However, the information contained in a single data source is usually difficult to meet the needs of accurate prediction. To this end, some studies have attempted to integrate multi-source information, give full play to the advantages of various network structures, and build large-scale complex network architectures to mine complex spatiotemporal correlation patterns in traffic flow data. These methods have indeed improved the prediction accuracy to a certain extent. However, the increase in model complexity will increase the training cost and computing resource requirements of the model, and ultimately affect the efficiency and scalability of practical applications (Lu and Osorio, 2018; Ji et al., 2022; Berghaus et al., 2024). Therefore, how to build an efficient and accurate traffic forecasting model is still one of the key issues that need to be overcome in the field of short-term traffic forecasting, and it is also the research goal of this paper.

**Table 1:** Summary of research on short-term traffic forecasting.

References	Basic model	Prediction target	Input	Dataset	Accuracy
Tian and Pan (2015)	LSTM	Volume	Volume	PeMS	MAPE = 6.49%
Zhao et al. (2017)	LSTM	Volume	Volume	Proprietary dataset	MRE = 6.41%
Yu et al. (2017)	LSTM	Speed	Speed and accident data	Proprietary dataset	MAPE = 1.03%
Liu et al. (2017)	LSTM CNN	Volume	Traffic network graph, speed and volume, …	PeMS	MAE = 4.41 MAPE = 6.99% RMSE = 6.42
Cai et al. (2020)	GCN Transformer	Speed	Traffic network graph, speed and volume	METR-LA	MAE = 2.43 MAPE = 4.73
				PeMS	MAE = 1.22 MAPE = 2.78
Yan et al. (2021)	Transformer	Speed	Speed, time of day, and day of the week…	METR-LA	MAE = 2.66 MAPE = 5.11% RMSE = 6.75
				Urban-BJ	MAE = 4.34 MAPE = 6.40% RMSE = 16.67
				Ring-BJ	MAE = 2.31 MAPE = 4.15% RMSE = 6.08
Ma et al. (2022)	CNN GRU	Speed	Speed	Proprietary dataset	MAE = 3.48 MAPE = 8.60% RMSE = 5.09
Chen et al. (2022)	DHM Transformer	Speed	Speed, volume time of day, and day of the week…	PeMSD3	MAE = 15.30 MAPE = 15.46% RMSE = 25.80
				PeMSD4	MAE = 18.53 MAPE = 12.37% RMSE = 29.96
				PeMSD7	MAE = 20.28 MAPE = 8.50% RMSE = 33.24
				PeMSD8	MAE = 13.58 MAPE = 9.21% RMSE = 23.08
Wang and Susanto (2023)	CNN LSTM	Volume	Traffic scene images, vehicle type, holidays, and weather	Proprietary dataset	MAE = 16.50 MSE = 0.50 RMSE = 22.26
Fang et al. (2023)	LSTM MCC	Volume	Volume	Amsterdam traffic dataset	MAPE = 11.57% RMSE = 280.87
Gong et al. (2023)	RGCN	Volume	Spatial knowledge graph and volume	Shanghai dataset	MAE = 0.15 RMSE = 30.22
				Nanjing dataset	MAE = 0.19 RMSE = 0.28

## 3 Methodology

### 3.1 Structure of Trafficformer model

**Figure 1:**
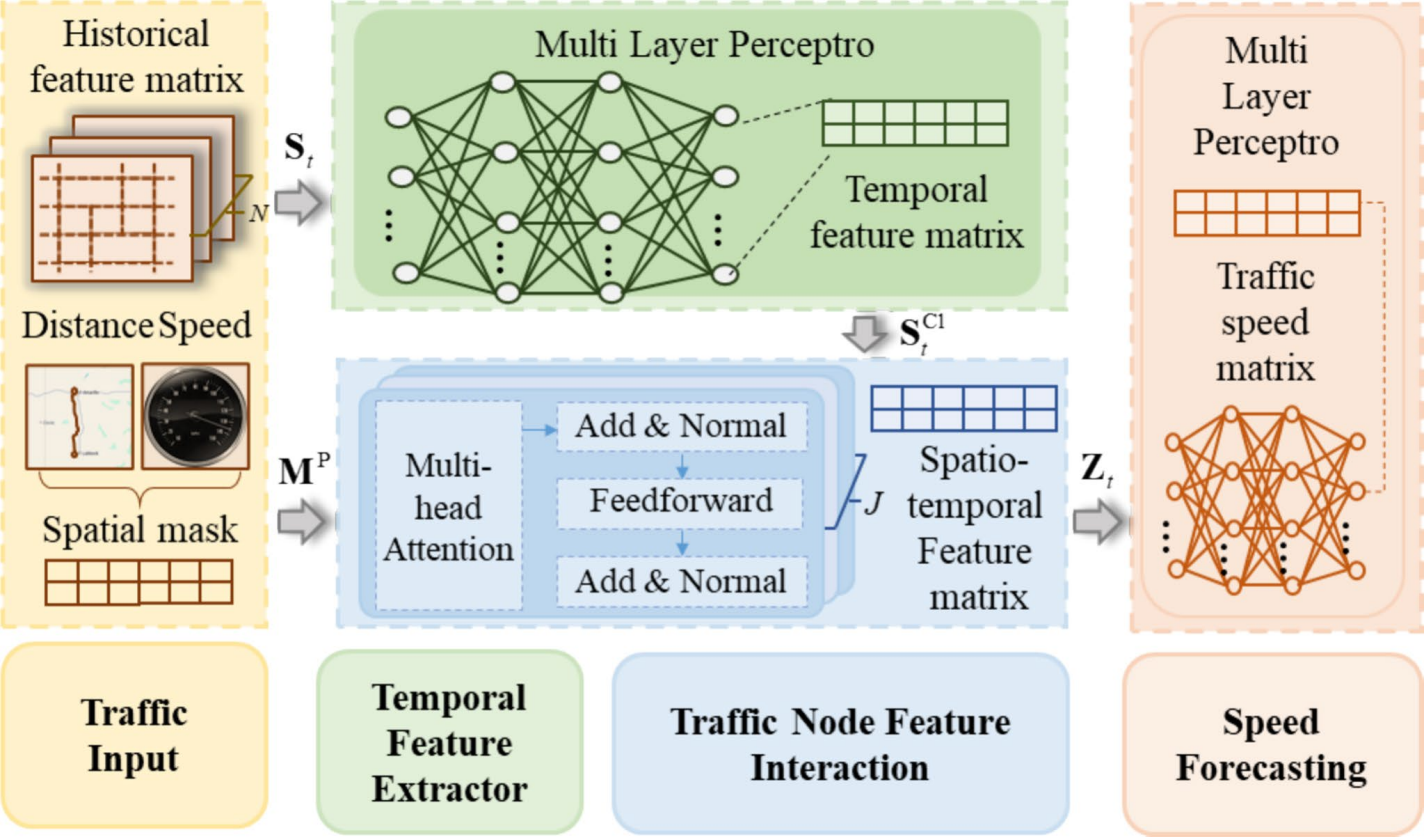
Structure of Trafficformer model.

The Trafficformer model introduced in this paper is designed for short-term traffic speed prediction at road network nodes, where traffic nodes represent the locations of traffic sensors on the road network. Figure 1 shows the structure of Trafficformer. As shown in Figure 1, the input of the model is the feature matrix $S_{t}\in {\mathbb{R}}^{I\times N}$ consisting of the traffic speeds of *N* consecutive steps of *I* nodes and the spatial mask $M^{P}\in {\mathbb{R}}^{I\times I}$ calculated by the node distance and free flow speed. Among them, the feature matrix $S_{t}$ is input into the traffic temporal feature extraction module, and the output is the matrix $S_{t}^{C1}\in {\mathbb{R}}^{I\times N}$ containing the traffic flow time series features. As *a priori* knowledge, $M^{P}$ specifically guides the model to focus on those nodes that are more likely to affect each other in space, so that the model can focus on the key spatial relationship faster and improve the prediction performance. With $S_{t}^{C1}$ and $M^{P}$ as input, the model realizes the extraction and embedding of spatial features based on the feature interaction module, and outputs the global feature matrix $Z_{t}\in {\mathbb{R}}^{I\times H}$ containing the spatiotemporal correlation of traffic flow. Finally, with $Z_{t}$ as input, the predicted speed matrix of each node can be obtained through the speed prediction module. Below, the three modules in the model will be elaborated on in detail.

### 3.2 Traffic node temporal feature extractor

The Temporal Feature Extractor for traffic nodes primarily consists of an MLP. MLP is a type of feedforward artificial neural network comprised of multiple layers of nodes. Each layer is fully connected to the next layer, and all nodes except the input nodes are neurons with non-linear activation functions. The use of activation functions introduces non-linearity to the output of the neurons, enabling MLP to handle non-linear separable problems effectively. Therefore, MLP is suitable for extracting temporal features with high uncertainty and non-linear characteristics. In this paper, the temporal feature extraction module for traffic nodes is a two-layer perceptron structure. It takes a feature matrix $S_{t}$ as input composed of the historical speeds of traffic flow of *I* nodes over a continuous sequence of *N* statistical intervals starting from time *t*. The feature matrix undergoes two neural network linear layers, one normalization layer, and one non-linear layer successively, ending up with a temporal feature matrix $S_{t}^{C1}$ that contains temporal information for each node, as shown in Equations 1–4.


(1) $$S_{t}^{Lin1}=S_{t}W^{Lin1}+b^{Lin1}\text{,}$$

where $S_{t}^{Lin1}\in {\mathbb{R}}^{I\times H}$ is the output of the first neural network linear layer (*H* refers to the hidden layer dimensions of the temporal feature extractor of traffic nodes); $W^{Lin1}\in {\mathbb{R}}^{N\times H}$ and $b^{Lin1}\in {\mathbb{R}}^{I}$ are learnable weight matrices, respectively.

To improve the accuracy of non-linear feature extraction and alleviate overfitting issues, a standardization layer and a non-linear layer have been introduced after the first linear layer. The standardization layer employed in this module is LayerNorm (Lei Ba et al., 2016). LayerNorm performs individual data sample training without relying on other data, which effectively avoids stability issues caused by the uneven distribution of mini-batch data in the batch normalization process during batch training. Furthermore, it eliminates the need to store mini-batch mean and variance and saves storage space. Considering the convergence speed of the model, the non-linear layer uses the ReLU activation function.


(2) $$S_{t}^{\mathrm{Lay}}=\mathrm{LayerNorm}\left(S_{t}^{Lin1}\right)\text{,}$$

(3) $$S_{t}^{\mathrm{ReLU}}=\mathrm{ReLU}\left(S_{t}^{\mathrm{relu}}\right)\text{,}$$

(4) $$S_{t}^{C1}=S_{t}^{\mathrm{ReLU}}W^{Lin2}+b^{Lin2}\text{,}$$

where $W^{Lin2}\in {\mathbb{R}}^{H\times H}$ and $b^{Lin2}\in {\mathbb{R}}^{I}$ are learnable weight matrices, respectively.

### 3.3 Traffic node feature interaction

Based on the traffic node temporal feature extractor, the temporal feature of each node was obtained. However, the spatial features among the nodes remained unprocessed. Therefore, subsequent to the traffic node temporal feature extractor, the traffic node feature interaction module was constructed using the encoder in the Transformer. The input of this module is $S_{t}^{C1}$ , which encompasses the temporal features of all nodes, and the output is the global feature matrix $Z_{t}$ that contains the spatiotemporal features of the nodes. The traffic node feature interaction module is constituted by *L* fundamental units. Each of these fundamental units mainly consists of a multi-head attention layer and a feedforward part. Among them, the multi-head attention layer is utilized to capture the complex spatial correlations and dependencies between different nodes by computing attention weights for each node’s features and generating new representations based on the weighted sum of other nodes’ features. And the feed-forward layer is employed to perform a non-linear transformation on the features obtained from the multi-head attention, mapping the input temporal feature matrix to the spatiotemporal feature output. It helps to further refine and enrich the feature representation, endowing the model with stronger discriminative ability. Next, a detailed introduction to the structures of the multi-head attention layer and the feedforward layer will be provided.

#### 3.3.1 Multi-head attention layer

The multi-head attention mechanism, which is an evolved form of the self-attention mechanism, functions by concurrently executing multiple self-attention heads. This parallel operation empowers the mechanism to capture the intricate dependency relationships within traffic node feature sequences from various vantage points, thereby endowing the traffic flow prediction model with more elaborate and accurate feature representations. In the context of each individual self-attention head, the model first derives the query, key, and value feature matrices that correspond to the node’s feature vectors. Subsequently, the model computes the attention weights between nodes by leveraging the query matrix of a particular node and the key matrices of other nodes. Finally, through the utilization of the value matrices of other nodes and their respective attention weights, the model achieves the update of the node feature matrix. Equations 5–9, with the *j*-th ( j=1,2,…,J ) self-attention head serving as a representative example, illustrate the update process of the feature matrix $Z_{t}^{j}\in {\mathbb{R}}^{I\times H}$ at time *t*.


(5) $$Q_{t}^{j}=S_{t}^{C1}W^{j,Q}\text{,}$$

(6) $$K_{t}^{j}=S_{t}^{C1}W^{j,K}\text{,}$$

(7) $$V_{t}^{j}=S_{t}^{C1}W^{j,V}\text{,}$$

(8) $$A_{t}^{j}=Q_{t}^{j}{\left(K_{t}^{j}\right)}^{T}\text{,}$$

(9) $$Z_{t}^{j}=\mathrm{softmax}\left(\frac{A_{t}^{j}}{\sqrt{d_{k}}}\right)V_{t}^{j}\text{,}$$

where $\left[Q_{t}^{j},\;K_{t}^{j},\;V_{t}^{j}\right]\in {\mathbb{R}}^{I\times H}$ are the query, key, and value feature matrices in the *j*-th self-attention head respectively; $W^{j,Q},\;W^{j,K},\;W^{j,V}\in {\mathbb{R}}^{H\times H}$ are the weight matrices, which can be updated during the training process; $A_{t}^{j}\in {\mathbb{R}}^{I\times I}$ is the attention weight in the *j*-th self-attention head; $\mathrm{softmax}\left(\cdot \right)$ is a normalization function that scales the values of each element in the matrix between 0 and 1 by dividing the attention weights between nodes by the sum of the weights; $d_{k}$ is a scaling factor primarily used to mitigate the gradient disappearance issue introduced by the softmax function, which is numerically equal to the dimension *H* of the row vector $k_{t}^{i,j}$ of the node keys in the matrix $K_{t}^{j}$ .

Theoretically, the self-attention mechanism possesses the capacity to incorporate the information of all nodes for the generation of a comprehensive feature matrix. Nevertheless, in real-world applications, especially when confronted with complex traffic networks that encompass a large number of nodes, if the model were to compute the attention weights with respect to all nodes without discrimination, it would entail exorbitant computational overheads and might introduce a significant amount of superfluous noise and interference. In light of this, prior information has been elected to be employed to fabricate a spatial mask $M^{P}$ . This mask allows the model to ignore nodes that are less likely to be relevant spatially when calculating attention weights. This effectively narrows the computational scope, reduces the impact of noise, and ultimately enhances both training efficiency and model accuracy. To be more specific, initially, the travel time expended by a vehicle in traversing each node at the free flow speed $V^{F}$ is computed. Here, the free flow speed pertains to the velocity at which a vehicle travels under an ideal, unimpeded traffic flow scenario. Subsequently, by considering the connectivity traits among the nodes within the road network, those nodes whose travel time falls within the range of [0, $T^{\mathrm{Limit}}$ ] are designated as strongly correlated nodes, while those with a travel time exceeding $T^{\mathrm{Limit}}$ are classified as weakly correlated nodes. The mask elements corresponding to the strongly correlated nodes are assigned a value of 1, and those corresponding to the weakly correlated nodes are set to 0. This process culminates in the construction of the spatial mask. Equations 10, 11 takes node *i* and node $i^{*}$ ( $i,\;i^{*}=\left[1,\;2,\ldots ,I\right];\;i\neq i^{*}$ ) as examples to illustrate the calculation process of the spatial mask.


(10) $$m^{i,i^{*}}=\left\{\begin{array}{l} 0\;\mathrm{if}\;T^{i,i^{*}}\leq T^{\mathrm{Limit}}\\ 1\;\mathrm{else}\;T^{i,i^{*}}>T^{\mathrm{Limit}} \end{array}\right.\text{,}$$

(11) $$T^{i,i^{*}}=\frac{L^{i,i^{*}}}{V^{F}}\text{,}$$

where $L^{i,i^{*}}$ is the actual distance between nodes, mile.

At this stage, the calculation methodology for the attention weight $A_{t}^{j}$ is revised as Equation 12:


(12) $$A_{t}^{j}=Q_{t}^{j}{\left(K_{t}^{j}\right)}^{T}⊗M^{P}\text{,}$$

where ⊗ denotes elementwise multiplication of matrices.

**Figure 2:**
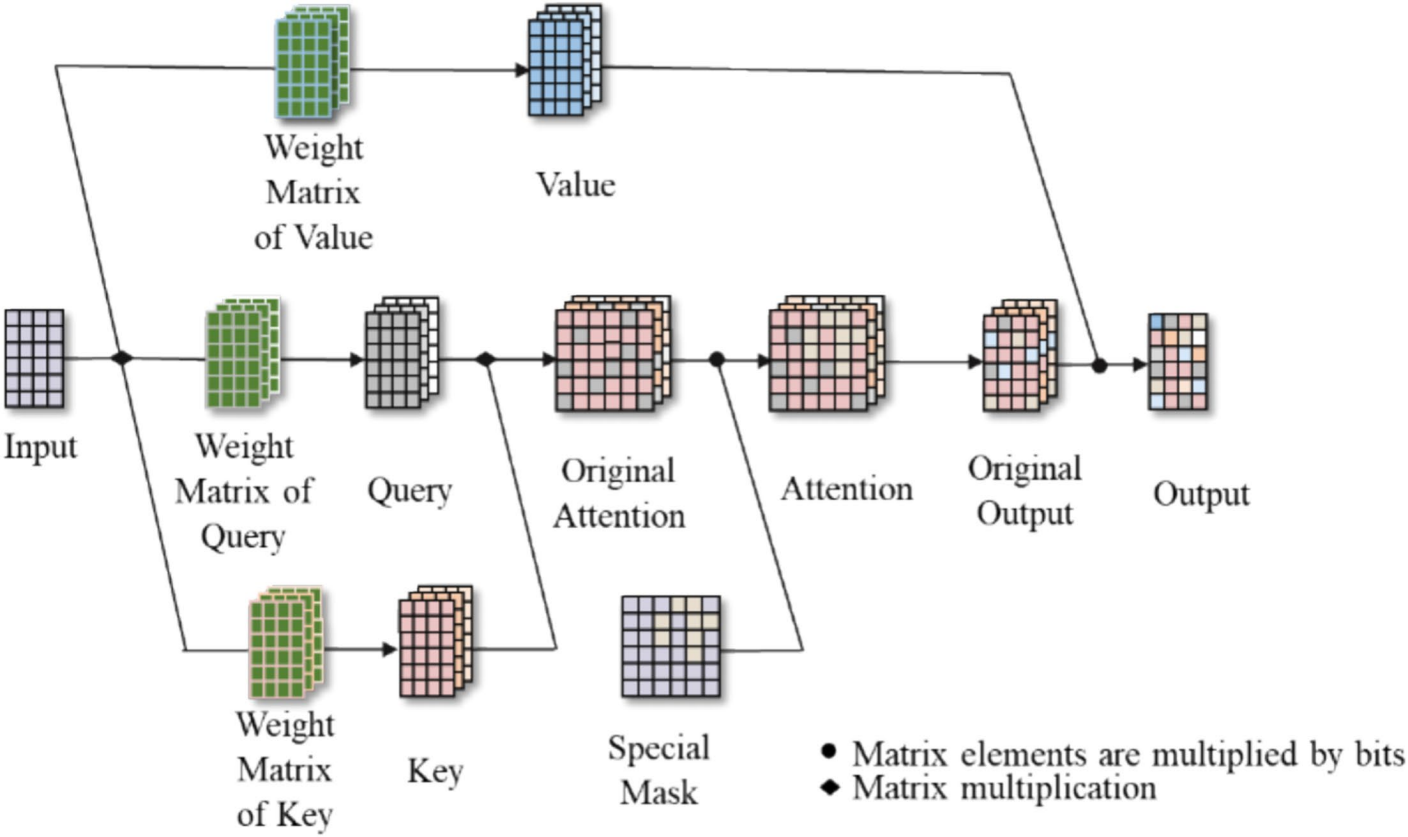
Structure of multi-head attention.

Once the feature matrix of each attention head have been computed, the global feature matrix $Z_{t}$ within the framework of the multi-head attention mechanism can be calculated in accordance with Equation 13. The multi-head attention mechanism’s network structure is presented in Figure 2.


(13) $$Z_{t}=\mathrm{Concat}\left(Z_{t}^{1},\;Z_{t}^{2},\ldots ,Z_{t}^{J}\right)W_{t}^{O}\text{,}$$

where $\mathrm{Concat}\left(\cdot \right)$ represents the concatenation operation, which specifically refers to horizontal concatenation of the feature matrices under different conditions in this paper; $W_{t}^{O}\in {\mathbb{R}}^{\left(H\times J\right)\times H}$ is a learnable weight matrix that represents the importance of different attention angles based on a global perspective.

#### 3.3.2 Feedforward networks

The feedforward network is a two-layer MLP structure. Unlike the normalization operation embedded within the traffic node’s temporal feature extraction component, the normalization operation in the feature interaction component is implemented separately by an external module. Therefore, the feedforward network consists only of fully connected layers and non-linear activation functions, as shown in Equation 14:


(14) $$F_{t}=\mathrm{ReLU}\left(Z_{t}W^{F1}+b^{F1}\right)W^{F2}+b^{F2}\text{,}$$

where $\left[W^{F1},W^{F2}\right]\in {\mathbb{R}}^{H\times H}$ , $\left[b^{F1},\;b^{F2}\right]\in {\mathbb{R}}^{I}$ are learnable weight matrices, respectively.

**Figure 3:**
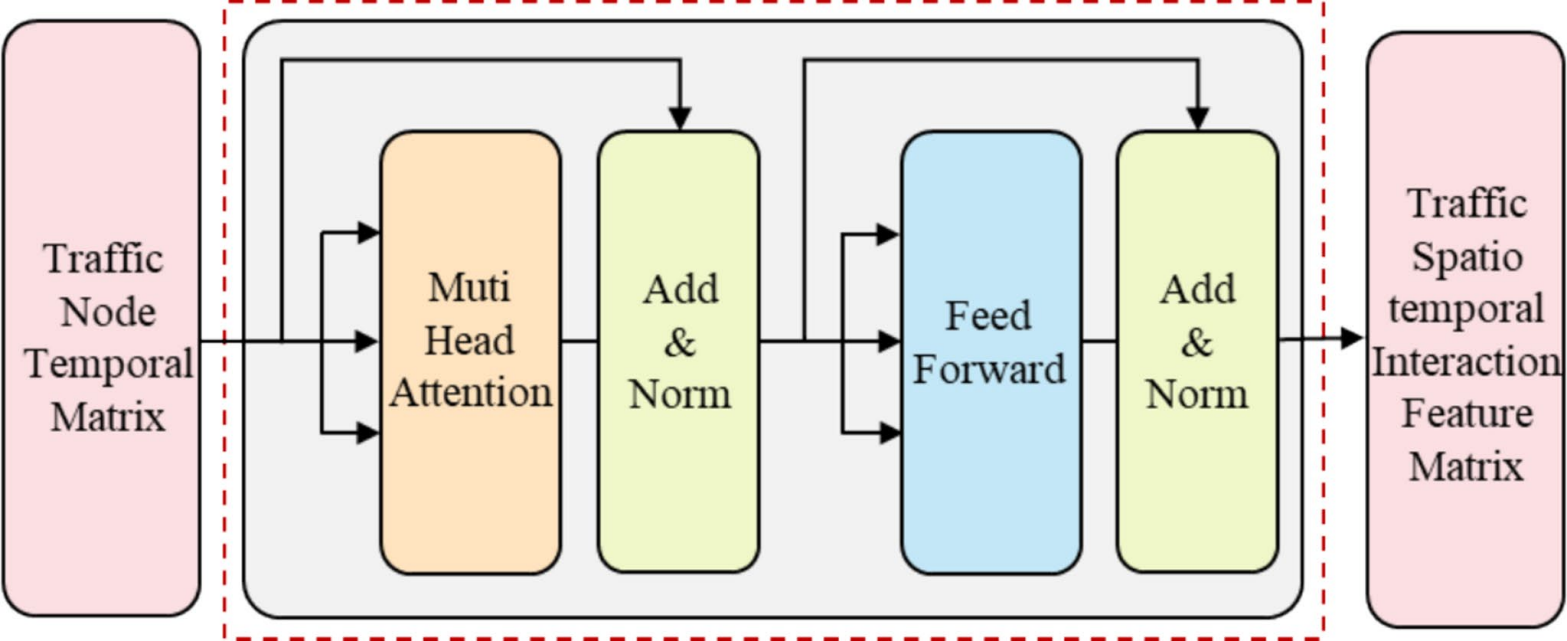
Feature interaction module structure of traffic nodes.

To build a deep model that effectively captures the complex spatiotemporal features in traffic flow data, Transformer employs residual connections around each module, followed by layer normalization, as shown in Equations 15, 16. In summary, the basic unit of the traffic node interaction module can be abstracted as the following equation, and the structure of the basic interaction module can be represented by Figure 3.


(15) $$Z_{t}^{C1}=\mathrm{LayerNorm}\left(Z_{t}+S_{t}^{C1}\right)\text{,}$$

(16) $$Z_{t}^{C2}=\mathrm{LayerNorm}\left(F_{t}+Z_{t}^{C1}\right)\text{,}$$

### 3.4 Traffic node speed forecasting

The traffic node speed forecasting module also follows the MLP structure, which is identical to the traffic node temporal feature extraction module. Both modules consist of two neural network linear layers, one normalization layer, and one non-linear layer. The difference lies in the input, output, and hidden layer dimensions of the network. The input of the traffic node speed forecasting module is the fused interaction feature matrix $Z_{t}^{C2}$ that captures the spatiotemporal correlations in the road network, while the output is the traffic speed matrix $S_{t}^{C2}\in {\mathbb{R}}^{I}$ for each node on road network at time step t+N+1 , as shown in Equations 17–20. MLP has various advantages of structure simplicity and highly parallel processing, which makes it computationally efficient for large-scale traffic forecasting tasks. This is why MLP has been chosen multiple times in this study for processing traffic node features.


(17) $$S_{t}^{C2,Lin1}=Z_{t}^{C2}W^{C2,Lin1}+b^{C2,Lin1}\text{,}$$

(18) $$S_{t}^{C2,\mathrm{Lay}}=\mathrm{LayerNorm}\left(S_{t}^{C2,Lin1}\right)\text{,}$$

(19) $$S_{t}^{C2,\mathrm{ReLU}}=\mathrm{ReLU}\left(S_{t}^{C2,\mathrm{Lay}}\right)\text{,}$$

(20) $$S_{t}^{C2}=S_{t}^{C2,\mathrm{ReLU}}W^{C2,Lin2}+b^{C2,Lin2}\text{,}$$

where $W^{C2,Lin1}\in {\mathbb{R}}^{H\times H^{*}}$ , $b^{C2,Lin1}\in {\mathbb{R}}^{I}$ , $W^{C2,Lin2}\in {\mathbb{R}}^{H^{*}\times 1}$ and $b^{C2,Lin2}\in {\mathbb{R}}^{1}$ are all learnable weight matrices; $H^{*}$ denotes the dimensions of hidden layers in the traffic node speed forecasting module.

## 4 Experiments

### 4.1 Dataset description

**Figure 4:**
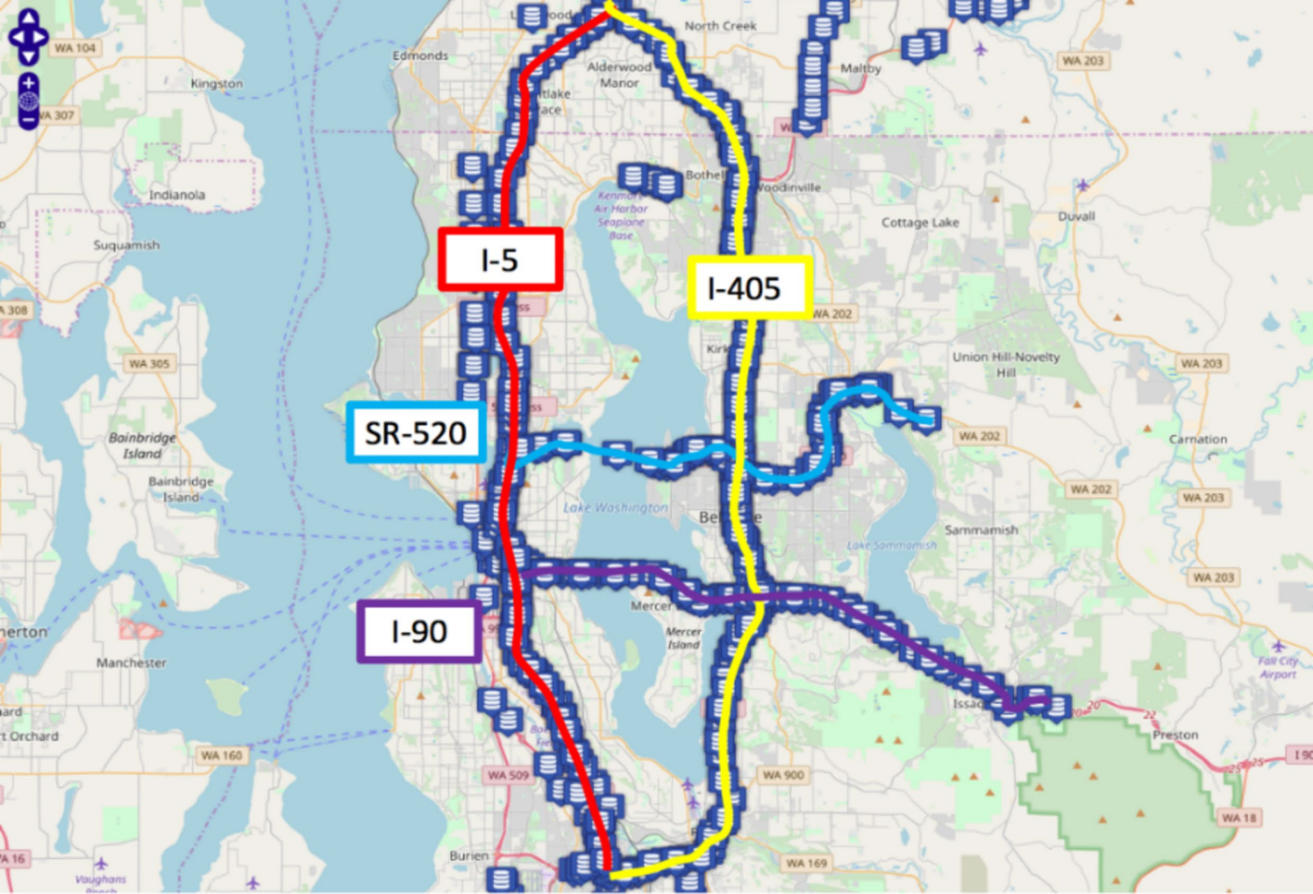
Seattle freeway satellite map (https://github.com/zhiyongc/Seattle-Loop-Data).

In this study, the efficacy of the method was evaluated by leveraging the publicly available Seattle Inductive Loop Detector Dataset V1 (referred to as the Loop dataset hereafter). This dataset consists of speed information collected from loop detectors deployed on four highways in the Seattle area: I-5, I-405, I-90, and SR-520. Each blue icon in Figure 4 represents a milepost on the road network, with a total of 323 mileposts along the entire route. For any given milepost, the speed information is obtained by averaging the data from multiple detectors on the corresponding main road direction. The dataset used in this study is available at the following link: https://github.com/zhiyongc/Seattle-Loop-Data.

The dataset contains the complete spatiotemporal speed information for the highway system in 2015, with a time interval of 5 min for each detector. The dataset comprises over 3.83 million records. In terms of the principle of algorithmic consistency, the model program was implemented based on the opensource code from a previous study (Cui et al., 2019). Several comparative experiments were performed using the identical dataset. The dataset was partitioned into three parts: training set, validation set, and test set, maintaining a 7:2:1 proportion. The training set served the purpose of model training, the validation set was reserved for finetuning and optimizing the parameters, and the test set was designated for evaluating the generalization performance of the model. Additionally, the road speed limit was set to 60 miles per hour, so $V^{F}=60$ mph is obtained. In the preprocessing stage, each speed value in the speed matrix is divided by the maximum speed value in the data set to normalize the speed data to the [0, 1] interval. This normalization operation is of great significance. It unifies the data scale, effectively improves the efficiency and stability of model training, and avoids the model’s excessive attention to certain features due to differences in data scale.

### 4.2 Experimental settings

#### 4.2.1 Baselines

In this paper, the Trafficformer model is compared with several established baseline models. These baseline models are carefully selected to represent a diverse range of techniques in the traffic flow prediction field, including both classic linear methods such as ARIMA and SVR, which possess well-established theoretical foundations but also come with certain limitations, and various nonlinear models like DiffGRU, LSTM, DMLP, LSTM+MLP, and TGG-LSTM. By comparing with these models, a thorough analysis of their performance is provided, and the distinct advantages of Trafficformer in different traffic forecasting scenarios are highlighted.

1. SVR: Support Vector Regression model (Hamed et al., 1995).
2. LSTM: Long Short-Term Memory network (Schmidhuber and Hochreiter, 1997).
3. ARIMA: Autoregressive Integrated Moving Average model (Smola and Schölkopf, 2004).
4. DiffGRU: An improved model based on Convolutional RNN. The spatial dependencies between traffic nodes are captured using Spectrogram Convolution, and the temporal dependencies are captured using enc-decoding components with scheduled sampling (Li et al., 2017).
5. TGG-LSTM: A DL model based on LSTM, which modeled the spatial correlations between different traffic nodes using graph convolution and utilized LSTM for vertical mining of the historical information of traffic flow (Cui et al., 2019).
6. DMLP: A network model consisting of two double-layered perceptions, where each MLP is responsible for traffic feature extraction and prediction, respectively (Wang Z. et al., 2024).
7. LSTM + MLP: A comparative algorithm proposed in relation to LSTM, aiming to highlight the unique significance of designing traffic flow feature extraction and prediction as separate modules. It consists of a single layer of LSTM for extracting traffic feature states and a two-layer perceptron for predicting traffic speed, which effectively improves the analysis of traffic flow data.

#### 4.2.2 Training parameters

All LSTM and MLP layers have the same weight dimensions, with a hidden layer size of 128. The input traffic flow data was composed of the historical speeds of traffic flow of 323 nodes over a continuous sequence of 10 artistical intervals starting from time *t*, denoted as N=10 . The predicted time step is 1. The size of the node connectivity constraint indicator $T^{\mathrm{Limit}}$ can be adjusted to observe the effects of feature extraction and interaction within different spatial ranges. Through multiple experiments, the value of $T^{\mathrm{Limit}}$ was set to 5. This means that each traffic node interacts with other traffic nodes that can be reached within 5 min of free flow speed from that node. Each model is trained with the goal of minimizing the MSE, which serves as a reliable and commonly used metric to quantify the disparity between the predicted and actual values. The optimization process is carried out using the AdamW optimizer, a sophisticated variant proposed by Loshchilov (2017). This optimizer ingeniously applies weight decay, a technique that effectively curtails the gradient of model parameters. By doing so, it not only mitigates the risk of overfitting but also substantially lowers the computational complexity associated with training. In terms of the learning rate strategy, the ReduceLROnPlateau approach (Ruder, 2016) has been adopted. This strategy is designed to dynamically adjust the learning rate based on the evaluation metrics. The initial learning rate is meticulously configured at 1E-3, a value determined through an extensive series of preliminary experiments. A decay factor of 0.2 is employed, which means that whenever the performance metric plateaus, the learning rate is reduced by this factor. The minimum learning rate is set at 1E-6 to ensure that the learning process does not stagnate completely. The total number of iterations is capped at a maximum of 150 to prevent excessive training and potential overfitting.

To further safeguard the convergence and generalization ability of the model, a mechanism to adaptively reduce the learning rate has been implemented. Specifically, if there is no observable improvement in performance for 10 consecutive epochs, the model will automatically reduce the learning rate. This adaptive learning rate adjustment strategy allows the model to finetune its learning pace and explore the parameter space more effectively, ultimately leading to better convergence and performance. In addition to the aforementioned strategies, a crucial regularization technique known as Early Stopping has been incorporated. The Early Stopping strategy acts as a safeguard against overfitting by closely monitoring the performance of the model on the validation set. Once the performance on the validation set ceases to improve, the training process is promptly halted. This ensures that the model is trained sufficiently to capture the underlying patterns in the data while preventing it from overfitting to the training data and losing its generalization capabilities. Overall, these meticulously designed optimization and regularization strategies work in tandem to enhance the performance, stability, and generalization ability of the model, enabling it to effectively handle the complex and dynamic nature of the traffic flow prediction task.

#### 4.2.3 Metrics

To evaluate the discrepancy between predicted traffic flow speed and actual traffic flow speed, three performance metrics are utilized: Mean Absolute Error (MAE), Mean Absolute Percentage Error (MAPE), and Root Mean Square Error (RMSE) (Li et al., 2021, 2022; Guo B. et al., 2024; Guo X. et al., 2024). The calculation method of the three metrics is shown in Equations 21–23.


(21) $$MAE=\frac{1}{I}\sum_{i=1}^{I}\left|{\hat{y}}_{i}-y_{i}\right|\text{,}$$

(22) $$\mathrm{MAPE}=\frac{1}{I}\sum_{i=1}^{I}\frac{\left|{\hat{y}}_{i}-y_{i}\right|}{y_{i}}\text{,}$$

(23) $$\mathrm{RMSE}=\sqrt{\frac{1}{I}\sum_{i=1}^{I}{\left({\hat{y}}_{i}-y_{i}\right)}^{2}}\text{,}$$

where, ${\hat{y}}_{i}$ represents the predicted speed of the traffic flow corresponding to node *i*, and $y_{i}$ represents the actual speed of the traffic flow corresponding to the same node, which serves as the data label.

### 4.3 Experimental results

#### 4.3.1 Comparative study

The performance metrics for each model on the test dataset can be found in Table 2. It can be observed that ARIMA and SVR are at a significant disadvantage. The limitations of these models stem from their inherent structural characteristics, which restrict their performance in large-scale prediction problems. For instance, ARIMA-based methods require the data to be stationary before making predictions, which can consume a significant number of computational resources in large-scale prediction tasks. Additionally, as mentioned in the Introduction, ARIMA-based methods have limited effectiveness in handling nonlinear data, which further restricts their applicability. While SVR performs well in handling low-dimensional and small sample datasets, it struggles with large-scale training samples and is sensitive to missing data. Consequently, it faces challenges in pre-processing and parameter tuning. On the other hand, DiffGRU and LSTM demonstrate a significant improvement in RMSE compared to ARIMA and SVR, with a reduction of 23%/26 and 53%/55%, respectively. This highlights the advantages of DL models in traffic forecasting. Traffic flow exhibits long-term fluctuations in both time and space, and these underlying patterns need to be mined and learned in the traffic forecasting process. Both GRU and LSTM leverage gate structures to achieve recurrent processing and feature extraction in sequential data. GRU does not have the forget gate structure found in LSTM, which may make it less effective in certain tasks requiring long-term dependencies. However, in some cases, GRU’s simplicity can lead to better efficiency. Furthermore, the network complexity of DiffGRU and LSTM is relatively low, and their ability to represent highly nonlinear road network features is limited with a small number of parameters. Therefore, their prediction accuracy is lower compared to other DL methods (models 5–8).

**Table 2:** Evaluation metrics of baseline model test set.

Number	Model	MAE/STD (mph)	MAPE (%)	RMSE (mph)
1	SVR	6.85/1.17	14.39	11.12
2	LSTM	2.70/0.18	6.83	4.97
3	ARIMA	6.10/1.09	13.85	10.65
4	DiffGRU	4.67/0.38	11.18	8.22
5	TGG-LSTM	2.57/0.10	6.01	4.63
6	DMLP	2.40/0.09	5.80	3.57
7	LSTM+MLP	2.40/0.09	5.70	3.56
8	Trafficformer	2.10/0.07	4.70	3.08

DMLP, LSTM+MLP, TGG-LSTM, and Trafficformer are four models with sufficient complexity to capture the nonlinear patterns within traffic flow data. Therefore, compared to the previous three models, all four models show a notable enhancement in accuracy. However, even the best performing model among the four, LSTM+MLP has a 16% higher RMSE compared to Trafficformer. The forecasting accuracy of the initial three models is similar but with some differences. DMLP and LSTM+MLP have the closest performance, indicating that a single-layer MLP and LSTM have similar effectiveness in extracting traffic flow features. Comparing them with a single-layer LSTM network also reveals the importance of designing separate networks for traffic flow feature extraction in improving prediction performance. TGG-LSTM takes into account the complex spatiotemporal features of traffic flow data and explores the prediction task thoroughly using LSTM and graph convolutional neural networks as core algorithms. Theoretically, it is supposed to surpass other DL algorithms that overlook traffic flow spatial features. However, its evaluation metrics are slightly higher than the other three algorithms. Relative to the proposed Trafficformer model, the MAE, MAPE, and RMSE show increases of 22, 27, and 50%, respectively.

The phenomenon can be explained by two main causes. First of all, the self-attention mechanism in Transformer permits the model to capture information from any position in the sequence, enabling better handling of long-range dependencies. On the other hand, GCN can only address long-range dependencies through expanding the number of convolutional layers. However, as the number of layers increases, the model’s effectiveness in capturing dependencies diminishes and the interpretability of the model is reduced. Therefore, prediction models based on GCN lack flexibility in feature extraction. Second, traffic data is typically collected by fixed location detectors at regular time intervals, resulting in sequences with clear temporal features. With the inherent advantages of attention mechanisms, Transformer can be applied to any type of input regardless of its shape. However, the GCN algorithm can only handle graph data, and treating traffic flow data as graph input disrupts the internal structure of the data to some degree, which limits the model’s performance and results in relatively lower accuracy. This does not mean that GCN-based network structures cannot be applied to traffic forecasting problems. When the data collection method changes, such as using image-based traffic data collected by video detectors, GCN-based models may achieve better prediction results (Li et al., 2024b,c).

In conclusion, the Trafficformer model shows significant improvements in MAE, MSE, and RMSE compared to other baseline methods, which indicates good performance in predicting future traffic flow.

In addition, to more rigorously evaluate the reliability of its performance improvements from a statistical perspective, LSTM + MLP, which performed best among the comparison methods, is selected. The predicted and true values from both models on the test set are used as inputs for paired *t*-tests and DM tests. The paired *t*-test is employed to determine whether there is a significant difference in the means of the two paired datasets. The null hypothesis states that the means of the two groups are equal, while the alternative hypothesis posits that the means are not equal. If the *p*-value obtained from the paired *t*-test is less than 0.05, the null hypothesis can be rejected, indicating a statistically significant difference between the means of the two groups. The DM test is used to compare whether there is a significant difference in the predictive accuracy of the two models. Its null hypothesis is that there is no difference in predictive accuracy between the two models, and the alternative hypothesis is that there is a difference (Iftikhar et al., 2023, 2024; Gonzales et al., 2024). When the *p*-value calculated from the DM test is less than 0.05, there is sufficient evidence to reject the null hypothesis, suggesting that the predictive accuracies of the two models differ significantly.

As shown in Table 3, the *p*-values from the paired *t*-tests between Trafficformer and LSTM+MLP are very small (averaging 3.27E-18 and 2.96E-03), well below the 0.05 significance level. Thus, the null hypothesis is rejected, confirming a statistically significant difference between the predicted and true values of the two models. Moreover, the DM test further supports this conclusion by rejecting the null hypothesis that the models’ predictive performances are identical. The multiple DM statistics and corresponding minimal *p*-values indicate that the prediction errors of the models are fundamentally different, reflecting the distinct effectiveness of their prediction mechanisms rather than random fluctuations. In summary, Trafficformer demonstrates clear advantages in both prediction accuracy and statistical significance, showcasing its broad application potential in traffic prediction problems.

**Table 3:** LSTM+MLP & Trafficformer statistical significance verification table.

Models			LSTM+MLP	Trafficformer
Paired *t*-tests	Step 1	*t*-statistic	6.03	10.96
		*p*-value	1.67E-09	8.74E-28
	Step 24	*t*-statistic	2.84	14.91
		*p*-value	4.55E-03	3.89E-47
	Step 123	*t*-statistic	2.85	8.59
		*p*-value	4.34E-03	9.82E-18
	Average	*t*-statistic	3.91	11.49
		*p*-value	2.96E-03	3.27E-18
DM tests	Step 1	DM statistic	22.70	
		*p*-value	0.00	
	Step 24	DM statistic	−8.38	
		*p*-value	1.55E-15	
	Step 123	DM statistic	2.53	
		*p*-value	0.01	
	Average	DM statistic	5.62	
		*p*-value	3.87E-3	

**Figure 5:**
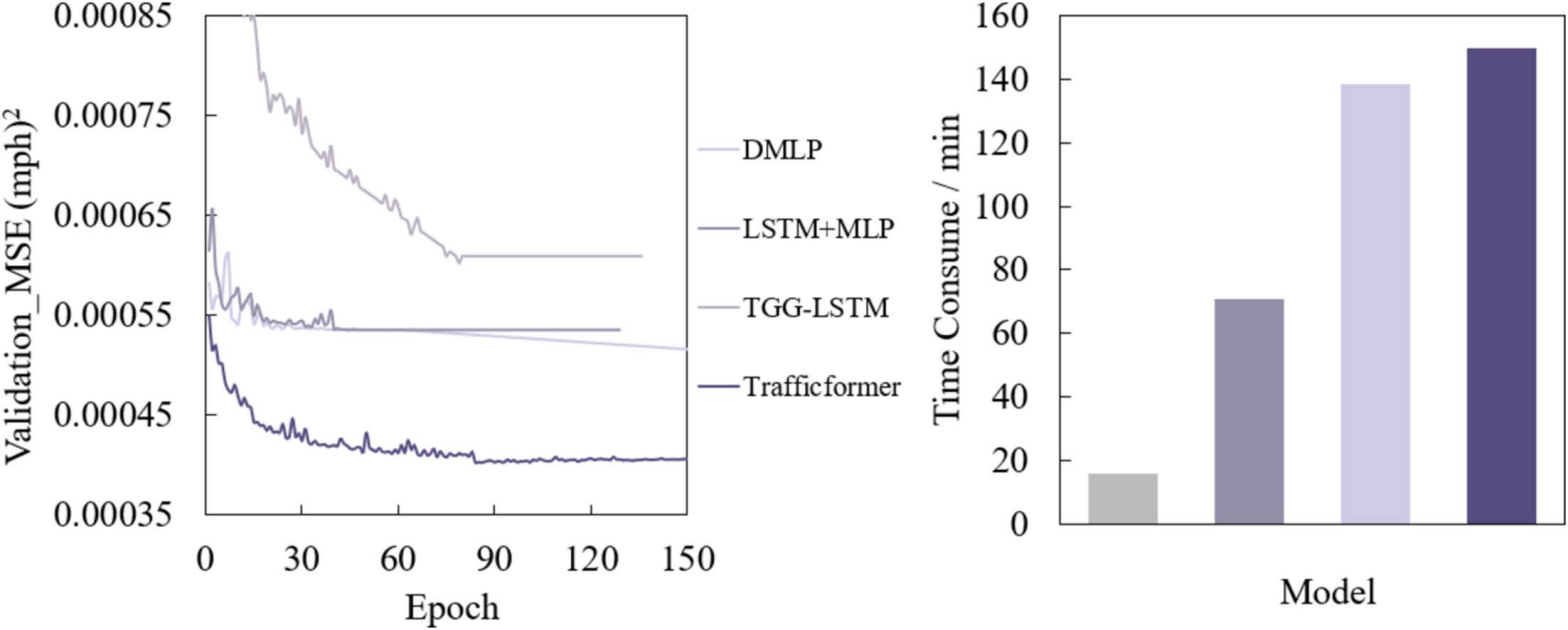
Mean square error of DL comparison model validation set and training time of DL comparison model training set.

Figure 5 shows the loss curves of the four deep neural network models on the validation set and the training time of DL comparison model training set.

Due to the introduction of early stopping, the number of iterations of each model during the training process is different. Interestingly, as the model complexity increases, the model training time gradually increases, which is opposite to the trend of model accuracy. DMLP and LSTM+MLP still show similar training time, and both models converge in about 50 epochs. TGG-LSTM converges in 84 epochs, while Trafficformer converges in 93 epochs. Figure on the right of Figure 5 shows the training time of the four algorithms on the training set at the same step size, from which similar conclusions can be drawn. It can be seen that relatively simple network architectures such as DMLP and LSTM+MLP are significantly faster in training than larger networks such as TGG-LSTM and Trafficformer. This shows that improving model accuracy comes at the cost of increasing training time. Therefore, in practical applications, it is necessary to balance accuracy and complexity according to specific scenarios and requirements. For scenarios where traffic flow patterns are relatively stable and have high real time requirements, simple models may have advantages due to their fast-computing speed and relatively simple deployment methods. For scenarios where traffic conditions are complex and changeable and have strict requirements on prediction accuracy, complex models have high training and deployment costs but can provide more accurate predictions and help with traffic management decisions.

#### 4.3.2 Ablation study

The Trafficformer model is a DL framework composed of three modules: traffic node feature extraction, traffic node feature interaction, and traffic node speed forecasting. The experimental data for models 3–6 in Table 2 have demonstrated the necessity of using separate feature extraction and prediction modules, underscoring the significant advantages of employing MLP as the feature extraction module in terms of accuracy and efficiency. With the other modules kept unchanged, this section focuses primarily on the analysis of the effectiveness of the node feature interaction module.

**Figure 6:**
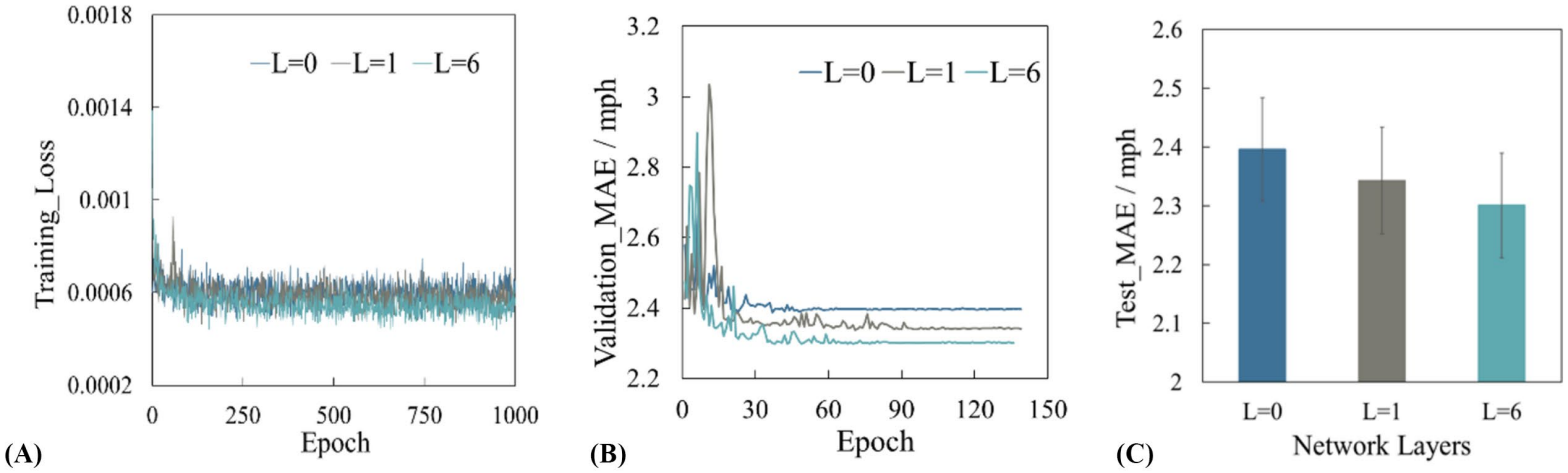
**(A)** Training loss curve, **(B)** validation set mean square error, **(C)** test set mean absolute error.

Figure 6 presents the performance of the models on the training, validation, and test sets when the number of layers within the module’s internal encoder represented by *L* varies (where *L* = 0 indicates the absence of the feature interaction module). It can be observed that as the number of encoder layers increases from 0, the performance of the model on the training set, validation set, and test set shows a trend of first rising and then stabilizing. This is because in the initial stage, increasing the number of encoder layers enables the model to gradually learn more complex spatiotemporal features and potential patterns in traffic flow data. The model achieves optimal performance when the number of encoder layers reaches 6. Therefore, this study sets the number of encoder layers in the interaction module to 6. In addition, it can be found that even without the spatial mask matrix based on road topology as *a priori* constraint, the performance of the model is still better after adding the interaction module. This is mainly due to the structural design inside the interaction module. The encoder in the interaction module can perform multi-level feature extraction and transformation on the input traffic node features, and enhance the model’s ability to learn complex relationships between nodes through information transmission and fusion between different layers. In addition, in each layer of the encoder, through the multi-head attention mechanism, the model can simultaneously focus on the correlation of different nodes in different feature subspaces, thereby capturing the dynamic change pattern of traffic flow in time and space dimensions.

**Figure 7:**
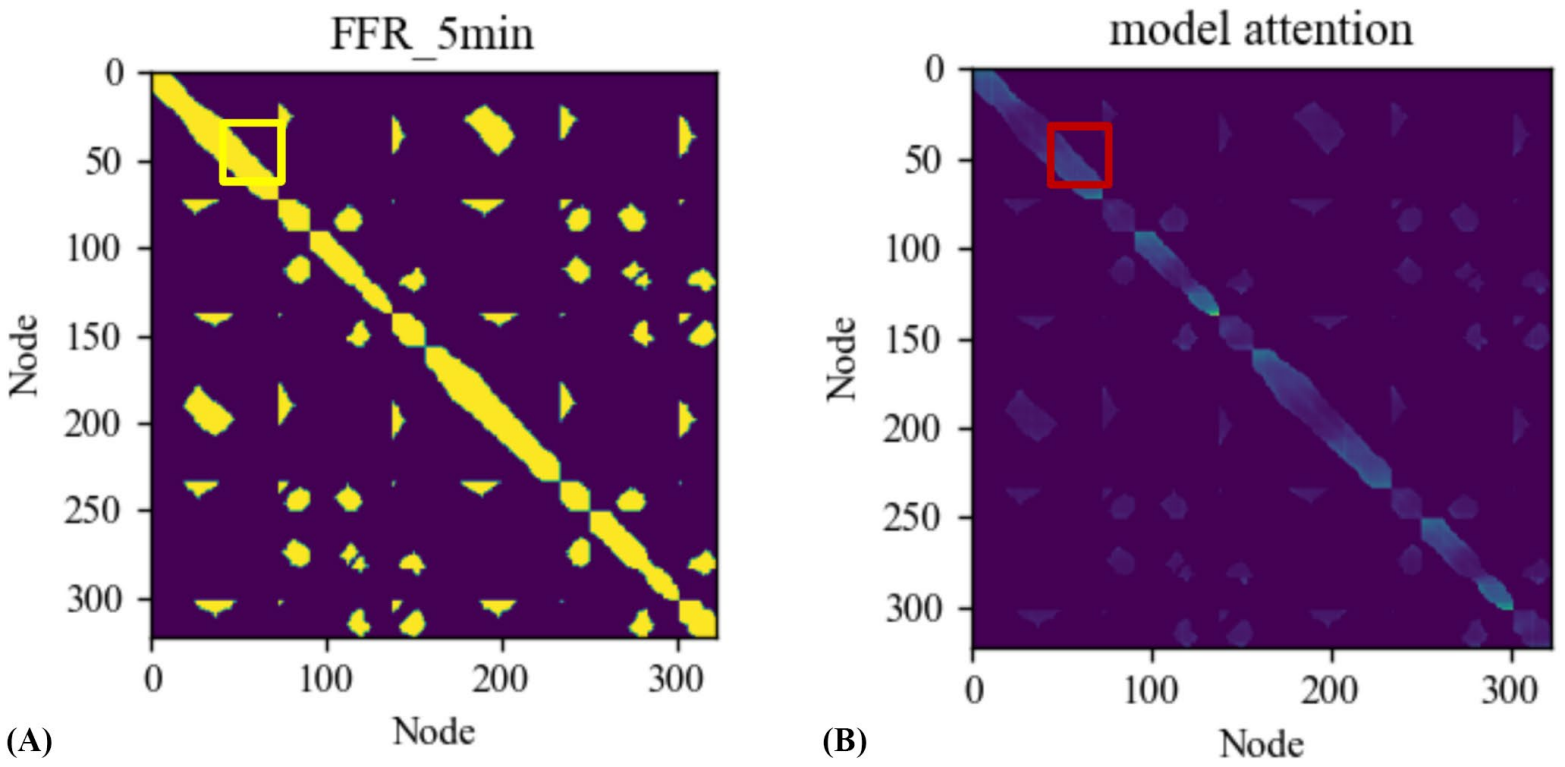
**(A)** The actual road network topology connectivity graph, **(B)** model attention value.

Furthermore, to better understand the effect of attention mechanism in the interaction module, this study plots the topological connectivity graph of the road network at using node indices as the x and y coordinates. As shown in Figure 7A, the yellow region represents the spatially connected target nodes. This connectivity does not imply the existence of roads for vehicle passage between the nodes but rather indicates the spatial range reachable by vehicles traveling at free flow speed. The spatial mask mentioned in the paper is also constructed based on this concept. Figure 7B displays the attention relationships between different nodes, where darker colors indicate stronger correlations between nodes. It can be observed that the learned attention of the Trafficformer model is within the range of the connectivity graph. Additionally, the darker regions in the graph mostly correspond to busy traffic segments as highway entrances or exits. Taking the location highlighted by the red box in Figure 7A as an example, it is a crossroad near the entrance of Mercer Island, located between I-90 and the city’s main arterial roads. This segment is a significant feature in the Loop dataset, and the dark markings within the yellow box in Figure 7B confirm this observation.

Based on the aforementioned analysis, this study introduces constraints based on road topology in both single-layer and multilayer interaction modules to investigate the importance of spatial masks. As shown in Table 4, for a network structure with only one interactive unit, after adding a spatial mask, the model’s prediction accuracy of node speed increased by 6.27, 9.34, and 10.41% on the training set, verification set, and test set, respectively. For a network structure with six interactive units, after adding spatial masks, the prediction accuracy of the model on the training set, validation set and test set increased by 33.95, 17.28 and 18.37%, respectively. Obviously, with the addition of spatial mask prior, the performance of the interaction module is significantly improved. This is mainly attributed to the optimization of the spatial mask in the model mechanism. From the perspective of interaction mode, it limits the range of interactive nodes, allowing the model to focus on highly accessible traffic nodes when calculating attention scores and feature fusion, avoiding interference from irrelevant nodes and accurately capturing influencing factors. From the perspective of information transfer, by discarding a large number of irrelevant node information, the model reduces the spread of redundant information during the training process, thereby significantly reducing the amount of calculation and improving the operating efficiency of the model. Therefore, the addition of spatial mask can enable the model to efficiently learn the spatial dependence in the traffic network, which is of key value in Trafficformer.

**Table 4:** Comparison model evaluation indexes.

Datasets and evaluation metrics		Single-layer interaction control group 1		Multilayer interaction control group 2	
		No spatial mask	With spatial mask	No spatial mask	With spatial mask
Training set	MSE (mph)^2^	5.44E-04	5.10E-04	5.76E-04	3.63E-04
Validation set	MAE (mph)	2.34	2.30	2.24	2.10
	MSE (mph)^2^	5.12E-04	4.64E-04	4.90E-04	4.05E-04
Test set	MAE (mph)	2.34	2.24	2.30	2.10
	MAPE (%)	5.50	5.10	5.40	4.70
	RMSE (mph)	3.50	3.31	3.41	3.08

**Figure 8:**
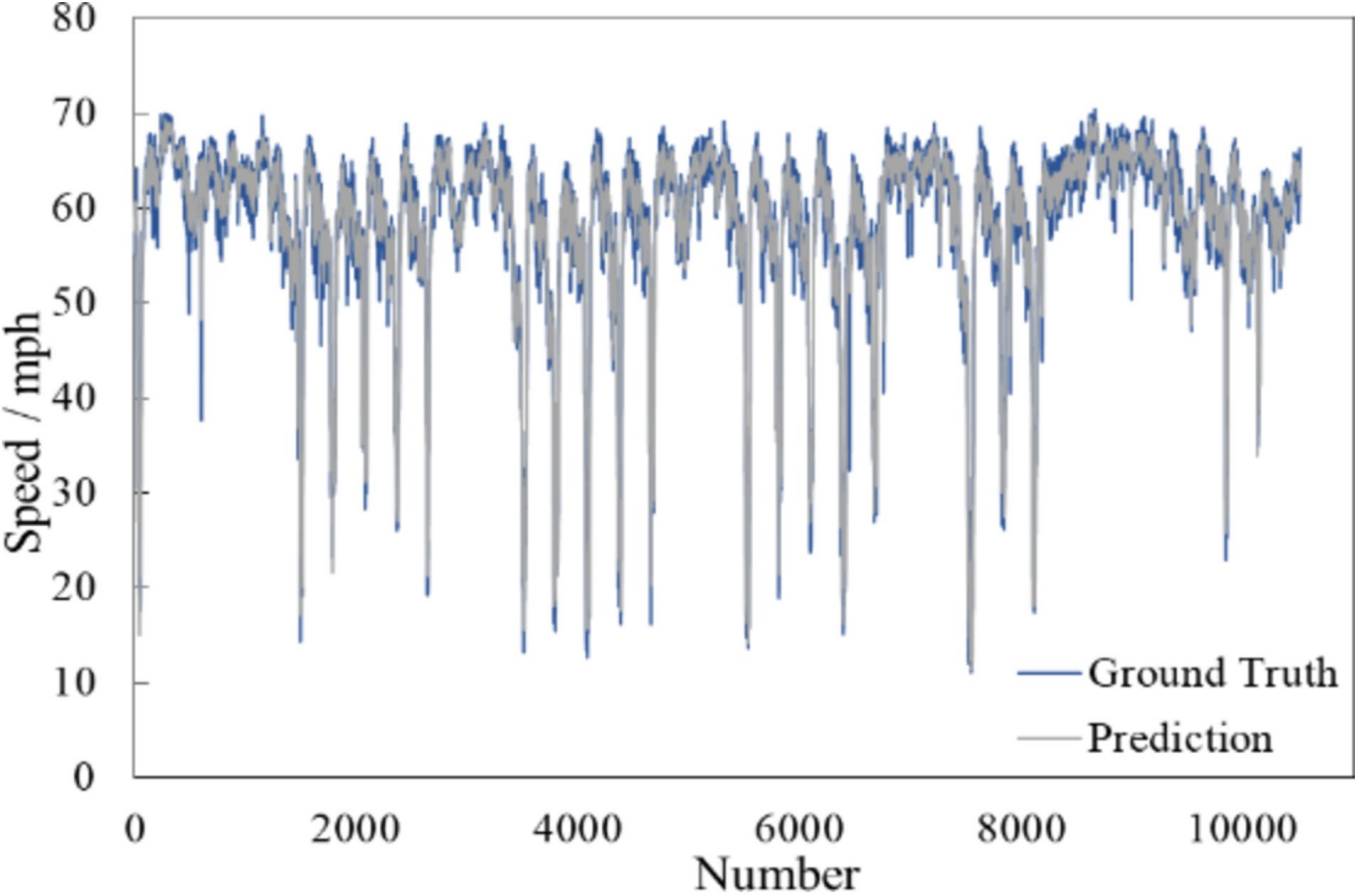
Example of traffic speed forecasting.

Figure 8 shows the comparison curves of the true value (blue curve) and the predicted value (grey curve) in the test set. It is apparent that, despite the traffic flow’s operating conditions, the predicted curve closely follows the actual curve. This observation indicates that the Trafficformer model is capable of effectively extracting traffic flow features and achieving high-precision predictions for spatiotemporal fused traffic networks.

## 5 Conclusion

In this paper, a DL framework built upon the Transformer architecture is proposed to address short-term prediction challenges in spatiotemporal fused traffic networks. Specifically, the multilayer perceptron and multi-head attention mechanisms are employed to efficiently extract spatiotemporal features of traffic flow. Prior constraints based on traffic node connectivity are also incorporated to limit interactions to reachable nodes, reducing unnecessary noise and improving both algorithm stability and precision. Test results demonstrate that the Trafficformer framework possesses a robust network structure and outperforms other baseline methods in both accuracy and computational complexity, making it particularly suitable for large-scale traffic forecasting tasks. In addition, using the learned attention distribution, managers can identify key traffic nodes and adjust control strategies accordingly, such as extending the green time of major roads or adjusting the signal phase of surrounding intersections, thereby optimizing traffic flow, alleviating congestion, and improving traffic efficiency.

Nevertheless, it is important to acknowledge the limitations of this paper. The model in this paper is mainly trained and predicted based on conventional traffic data. However, traffic flow is affected by many special factors such as weather, traffic accidents, and road construction. The model is not adaptable and flexible enough to these special situations, and the prediction accuracy will be reduced when encountering abnormal situations. In future work, more metadata, including but not limited to weather data, event report data, etc., will be introduced, and these special factors will be incorporated into the model training process. This aims to enhance the model’s ability to cope with various complex situations, thereby improving its prediction accuracy under abnormal conditions.

## Data Availability

The raw data supporting the conclusions of this article will be made available by the authors, without undue reservation.
